# Several Instances of the Good Effects of Opium in Mortifications

**Published:** 1784

**Authors:** 


III. *Several inftances of the good effects of Opium in mortifications. Communicated to Dr.* Simmons, F. R. S. *by* Robert Hamilton, M. D. *Phyfician at Ipfwich, Extra-Licentiate of the Royal College of Phyficians, London; and Member of the Medical Society of Edinburgh.*

MR. Pott was the firft, I believe, who recommended the ufe of opium in mortifications; but I do not know that it has yet come into general ufe among furgeons.

About a year ago I had occafion to experience its good effects in a patient labouring under a mortification in one of his legs, whom I vifited with Mr. Law, an ingenious furgeon at St. Alban's. In this cafe the Gaftrocnemius mufcle was almoft entirely confumed, and the difeafe was ftill increafing, depriving the patient of fleep by the excruciating pain that attended it. A hiccough alfo was become a troublefome and alarming fymptom at the time I firft faw him.

Under theſe circumſtances we had recourſe to opium, adminiſtering it in large quantities, and increaſing the doſe till reſt was procured. Its good effects ſoon became apparent; it ſpeedily put a ſtop to the farther progreſs of the diſeaſe, and in a ſhort time the patient was reſtored to health.

I have ſince experienced the efficacy of the ſame medicine in another caſe of a ſimilar nature. In this laſt caſe the patient began with taking one grain of opium every three hours, and by degrees increaſed the doſe to five grains. The day after he began the uſe of this remedy the pain which, from the mortification of his toes, had been conſtant and violent, began to abate, as did all the other ſymptoms, and it was not long before the ſphacelated parts began to ſeparate. To theſe two caſes I beg leave to add a third communicated to me lately by Mr. Law, the gentleman above-mentioned, and which I ſhall give in his own words, from his letter dated December 8th, 1783. "I have at "preſent," ſays he, "the earl of Bute's princi-"pal gardener under my care, with a mortifi-"cation in one of his toes, which was pre-"ceded by an almoſt intolerable burning pain "that continued from the 30th of Novem-"ber till this morning, when I had the ſatisfac-

"tion

" tion to hear that he had slept nine hours, and " that the hiccough, which was very dreadful, " had ceased. He is now free from pain, and " a line of separation begins to appear. During the last ten days, I gave him twenty " drops of *Tinctura Thebaica* in a decoction of " bark every three hours, with three grains of " strained opium, and at last I increased the " dose of the latter to seven grains, when I " gained my point." Such success as this may probably induce the medical practitioner to have recourse more frequently to this remedy in mortifications, especially as we sometimes see the bark fail.

Ipswich, March 10, 1784.

